# Computational Analysis Reveals a Key Regulator of Cryptococcal Virulence and Determinant of Host Response

**DOI:** 10.1128/mBio.00313-16

**Published:** 2016-04-19

**Authors:** Stacey R. Gish, Ezekiel J. Maier, Brian C. Haynes, Felipe H. Santiago-Tirado, Deepa L. Srikanta, Cynthia Z. Ma, Lucy X. Li, Matthew Williams, Erika C. Crouch, Shabaana A. Khader, Michael R. Brent, Tamara L. Doering

**Affiliations:** a Department of Molecular Microbiology, Washington University School of Medicine, St. Louis, Missouri, USA; b Center for Genome Sciences and Systems Biology, Washington University School of Medicine, St. Louis, Missouri, USA; c Department of Computer Science and Engineering, Washington University, St. Louis, Missouri, USA; d Department of Pathology and Immunology, Washington University School of Medicine, St. Louis, Missouri, USA; e Department of Genetics, Washington University School of Medicine, St. Louis, Missouri, USA

## Abstract

*Cryptococcus neoformans* is a ubiquitous, opportunistic fungal pathogen that kills over 600,000 people annually. Here, we report integrated computational and experimental investigations of the role and mechanisms of transcriptional regulation in cryptococcal infection. Major cryptococcal virulence traits include melanin production and the development of a large polysaccharide capsule upon host entry; shed capsule polysaccharides also impair host defenses. We found that both transcription and translation are required for capsule growth and that Usv101 is a master regulator of pathogenesis, regulating melanin production, capsule growth, and capsule shedding. It does this by directly regulating genes encoding glycoactive enzymes and genes encoding three other transcription factors that are essential for capsule growth: *GAT201*, *RIM101*, and *SP1*. Murine infection with cryptococci lacking Usv101 significantly alters the kinetics and pathogenesis of disease, with extended survival and, unexpectedly, death by pneumonia rather than meningitis. Our approaches and findings will inform studies of other pathogenic microbes.

## IMPORTANCE

*Cryptococcus neoformans* causes fatal meningitis in immunocompromised individuals, mainly HIV positive, killing over 600,000 each year. A unique feature of this yeast, which makes it particularly virulent, is its polysaccharide capsule; this structure impedes host efforts to combat infection. Capsule size and structure respond to environmental conditions, such as those encountered in an infected host. We have combined computational and experimental tools to elucidate capsule regulation, which we show primarily occurs at the transcriptional level. We also demonstrate that loss of a novel transcription factor alters virulence factor expression and host cell interactions, changing the lethal condition from meningitis to pneumonia with an exacerbated host response. We further demonstrate the relevant targets of regulation and kinetically map key regulatory and host interactions. Our work elucidates mechanisms of capsule regulation, provides methods and resources to the research community, and demonstrates an altered pathogenic outcome that resembles some human conditions.

## INTRODUCTION

*Cryptococcus neoformans* kills over 600,000 people each year (1) and causes up to 20% of AIDS-related deaths in developing areas of the world (2). This opportunistic fungal pathogen is ubiquitous in the environment and is contracted by the inhalation of spores or desiccated yeast cells, which leads to a primary pulmonary infection. Healthy individuals generally control the organism, although they likely continue to harbor latent infection. In the setting of immunocompromise, however, either at the time of initial contact or beyond, the yeast can grow and disseminate, with a particular predilection for the central nervous system. This tropism and the ability of the organism to cross the blood-brain barrier (BBB) result in cryptococcal meningitis, which is the most devastating manifestation of *C. neoformans* infection. This condition is fatal if not treated and is associated with significant mortality even in advanced health care settings (3).

A variety of characteristics have been implicated in *C. neoformans*’ success as a pathogen. These include factors that influence fungal survival in the host, such as survival at mammalian body temperature, ability to withstand the oxidative stress encountered in a host phagosome, and the production of degradative enzymes. The production of melanin, which increases cryptococcal resistance to environmental insults, also contributes to fungal virulence. The primary virulence factor of this pathogen, however, is the display of an elaborate polysaccharide capsule, which is required for virulence and is unique among the pathogenic fungi (4). Shed polysaccharides impede the host immune response (5), and the size of the capsule affects phagocytosis of *C. neoformans*; these in turn alter the balance of free and host cell-engulfed fungi, influencing key events, such as clearance, latency, and dissemination. Notably, capsule thickness changes dramatically in response to environmental conditions, mediated by signaling pathways that sense the host environment and detect nutrient limitation, increased temperature, and CO_2_ levels (6). To fully understand synthesis of this major virulence factor and how it is induced during infection to the detriment of the host, we are developing a complete and integrated model of capsule regulation.

To define the transcriptional network that regulates capsule, we have been applying computational and genomic tools (7, 8). Network analysis of gene expression data (using our NetProphet algorithm [9]) indicated that Usv101, a C_2_H_2_ transcription factor named for a *Saccharomyces cerevisiae* ortholog (see below), regulates multiple genes known to be required for capsule growth (8). The corresponding gene, *USV101*, had already come to our attention because its transcript abundance showed a significant negative correlation with capsule size across a range of capsule-inducing growth conditions (7) (see Fig. S1 in the supplemental material). Our PhenoProphet algorithm also predicted that Usv101 would be required for normal capsule regulation (8). Recently, Jung et al also termed Usv101 a core fungal transcription factor (TF), based on its conservation in model yeasts and fungi (10).

The expression pattern of *USV101* suggested that cells lacking this gene would be hypercapsular, a prediction that we confirmed using *usv101*Δ mutant strains (8). The mutant cells also showed markedly impaired growth in mouse lung, despite their large capsules and wild-type (WT) growth in rich medium (8). This was surprising because low virulence is generally associated with reduced capsule size. Here, we apply network analysis to mechanistically explain the mutant phenotypes of cells lacking this important TF and demonstrate that Usv101 influences not only multiple virulence factors, but also the course and outcome of cryptococcal infection.

## RESULTS

### Capsule induction requires new protein and RNA synthesis.

**FIG 1:**
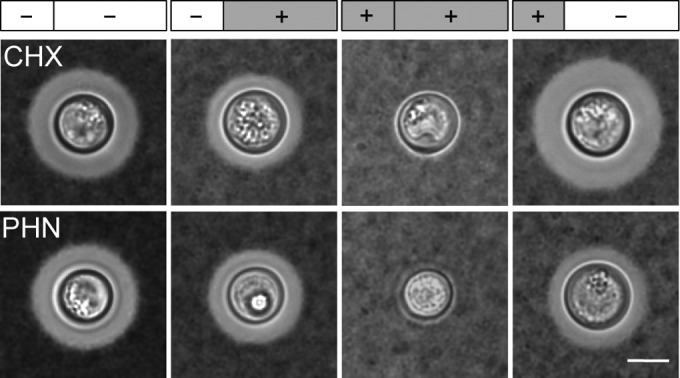
Capsule induction requires transcription and translation. Wild-type (KN99α) cells were grown under capsule-inducing conditions (DMEM, 37°C, 5% CO_2_) for 24 h, with (+) or without (−) cycloheximide (CHX) or phenanthroline (PHN). Drugs were present during growth for the first 8 h, last 16 h, both, or neither, as indicated on the bars above the images. Samples were stained with India ink, and light micrographs of representative cells are shown, all to the same scale (scale bar = 5 µm). Capsule thickness values for these strains are tabulated in Table S1A in the supplemental material.

Capsule might be regulated at multiple levels. These could include mechanisms involving transcription, mRNA (stability or localization), or posttranslational processes (such as modification or relocalization of proteins involved in capsule biosynthesis). To test whether new protein synthesis is required for capsule growth, we induced cells to enlarge their capsules in the absence or presence of cycloheximide (CHX), which inhibits protein synthesis by preventing translational elongation, and then measured capsule thickness. (Representative images are shown in Fig. 1, and population measurements are provided in Table S1A in the supplemental material.) Cells exposed to CHX throughout a 24-h period of capsule induction showed severely impaired capsule growth compared to untreated cells (Fig. 1, top row, compare first and third images); if CHX was introduced partway through induction (at 8 h), the cells generated capsules of the size expected from only the initial induction period (Fig. 1, top row, second image). Finally, the inhibition of capsule growth was reversible by the restoration of protein synthesis, as shown by cells treated for 8 h with CHX and then washed into medium without drug for the remaining 16 h of induction (Fig. 1, top row, fourth image). Notably, these cells displayed slightly larger capsules than untreated cells, consistent with a “rebound” effect potentially due to mRNA accumulation during CHX treatment. These results strongly suggest that new protein synthesis is indeed required for capsule enlargement, although they do not rule out indirect effects of CHX on secretory vesicles (11), which have been implicated in capsule synthesis (12).

Since capsule regulation occurs upstream of protein synthesis, it is likely to act at the transcriptional level. However, other upstream steps, such as regulation of translation, mRNA stability, or mRNA localization, might also be sufficient for capsule induction. To test whether new transcription is required, we treated cells with 1,10-phenanthroline (PHN), an inhibitor of RNA synthesis. The results with this compound (Fig. 1, bottom row) were qualitatively similar to those of the CHX experiment, except that treated cells restored to drug-free medium did not generate full-size capsules. This may be because inhibition of transcription is a more drastic insult to the cells, from which they are slow to recover. In any event, these studies support our working model that capsule synthesis requires both mRNA and protein synthesis, suggesting that the dominant regulatory influences on capsule occur at the level of transcription.

### Usv101 regulates multiple phenotypes, including major virulence traits

**FIG 2:**
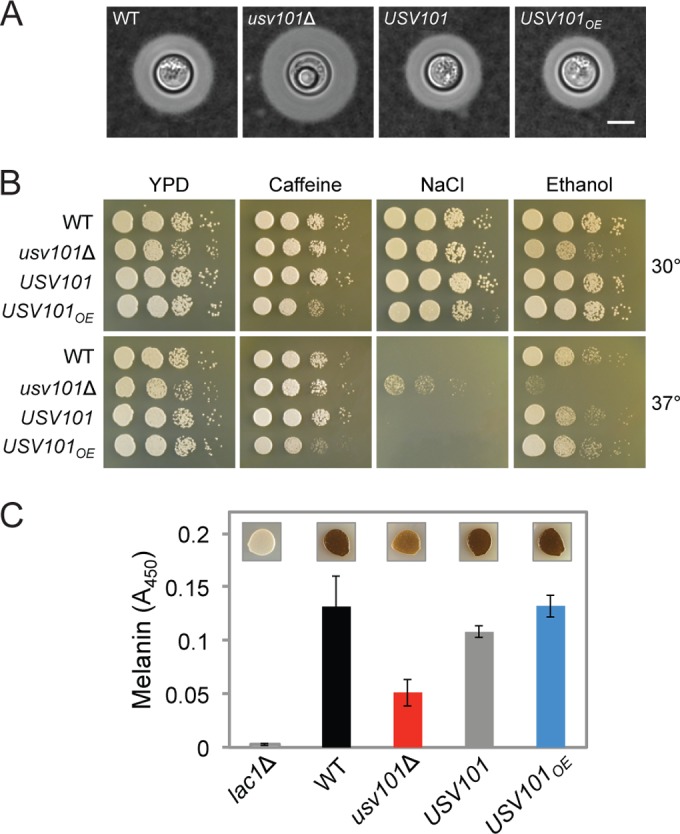
Capsule, growth, and melanization of *usv101* mutants. (A) India ink negative staining of wild type (WT), *usv101*Δ, complemented *usv101*Δ (*USV101*), and *USV101*-overexpressing (*USV101_OE_*) strains. Representative cells are shown, all to the same scale (scale bar = 5 µm). Capsule thickness values are tabulated in Table S1B in the supplemental material. (B) Ten-fold serial dilutions of the same strains grown on rich medium alone (YPD) or YPD with various stressors at the indicated temperatures. (C) The same strains were assessed for production of cell-associated melanin, which yields brown colonies (insets; 10^4^ cells spotted on l-3,4-dihydroxyphenylalanine [l-DOPA] medium and grown at 30°C), and for melanin released into the medium (measured by *A*_450_ [Materials and Methods]). A strain that cannot synthesize melanin due to absence of a key laccase (*lac1*Δ) is shown as a control.

Usv101 is predicted to be a DNA-binding protein. Consistent with this, a C-terminal hemagglutinin (HA)-tagged version of the protein localized to the nucleus by immunofluorescence microscopy, whether cells were grown in non-capsule-inducing (yeast extract-peptone-dextrose [YPD]) or capsule-inducing (Dulbecco’s modified Eagle’s medium [DMEM]) conditions (not shown). For studies of Usv101 function, we complemented our transcriptome sequencing (RNA-seq)-confirmed *usv101*Δ mutant (8) with the unmodified wild-type gene at the endogenous genomic locus (*USV101* strain); we also generated a Usv101-overexpressing (*USV101_OE_*) strain by replacing the endogenous promoter with that of *ACT1*. Negative staining showed that the hypercapsular phenotype of the *usv101*Δ mutant was corrected when the gene was complemented and that the capsule size of the overexpression strain was reduced compared to that of the wild type (Fig. 2A; see Table S1B in the supplemental material). Together, these observations support the action of Usv101 as a repressor of capsule expansion. Interestingly, the increase in *usv101*Δ capsule size is accompanied by a decrease in capsule polysaccharide that is shed into the medium, a trait we had observed earlier (8) and here quantitated for the mutant, complemented, and overexpressor strains (see Fig. S2A in the supplemental material).

*USV101* has two orthologs in *S. cerevisiae*, *USV1* (alias *NSF1*) and *RGM1*, which are paralogs of each other resulting from a whole-genome duplication along the *S. cerevisiae* lineage (13). The three genes are most similar in their C_2_H_2_ zinc finger DNA binding domains. Rgm1 is a TF that is involved in cell growth (14), activates genes involved in central carbon metabolism, and regulates expression of Y′ telomeric elements and subtelomeric genes (SGD project; http://www.yeastgenome.org/download-data/ [15 July 2015]). Usv1 is involved in energy metabolism and directly regulates genes in response to nutrient limitation conditions (15). It binds to promoter regions of genes involved in general responses to stresses, including heat shock, oxidative stress (16), and high salt (17). Consistent with this role, *S. cerevisiae* strains lacking *USV1* are sensitive to stresses, including ethanol, salt, and high concentrations of glucose (18–20). We next tested whether the cryptococcal Usv101 was also involved in the regulation of stress responses.

Although the *usv101*Δ mutant showed a subtle growth defect compared to the wild type on rich medium (YPD) at both 30 and 37°C, it grew normally under multiple conditions that challenge cell integrity (not shown), including media containing Congo red (0.5%), SDS (0.02%), sorbitol (1.5 M), or caffeine (0.05%) (Fig. 2B). Interestingly, although the *S. cerevisiae* usv1Δ mutant (17) is sensitive to salt, the *usv101*Δ mutant was more resistant than the wild type to high NaCl (1.2 M) at 37°C; the *USV101* overexpression strain also exhibited a subtle sensitivity to high salt (Fig. 2B). Finally, the *usv101*Δ mutant was sensitive to 6% ethanol (similar to the *S. cerevisiae* usv1Δ mutant [20]), and the overexpression (*USV101_OE_*) strain was sensitive to 0.05% caffeine (Fig. 2B).

We next tested the ability of the *usv101*Δ mutant and control strains to withstand nitrosative and oxidative stresses and produce melanin, traits that are associated with resistance to environmental stress and increased virulence in *C. neoformans* (21–23). Growth of the *usv101*Δ and *USV101_OE_* strains was not affected by H_2_O_2_ (not shown), but the deletion mutant was defective in melanin production, as shown by both colony pigmentation and quantitative assay (Fig. 2C). The *usv101*Δ mutant did show slight sensitivity to nitrosative stress compared to wild-type cells (see Fig. S2B in the supplemental material); this sensitivity was not corrected by melanin induction prior to spotting (see Fig. S2B [data not shown]).

### Usv101 is required for normal host-pathogen interactions *in vitro*.

**FIG 3:**
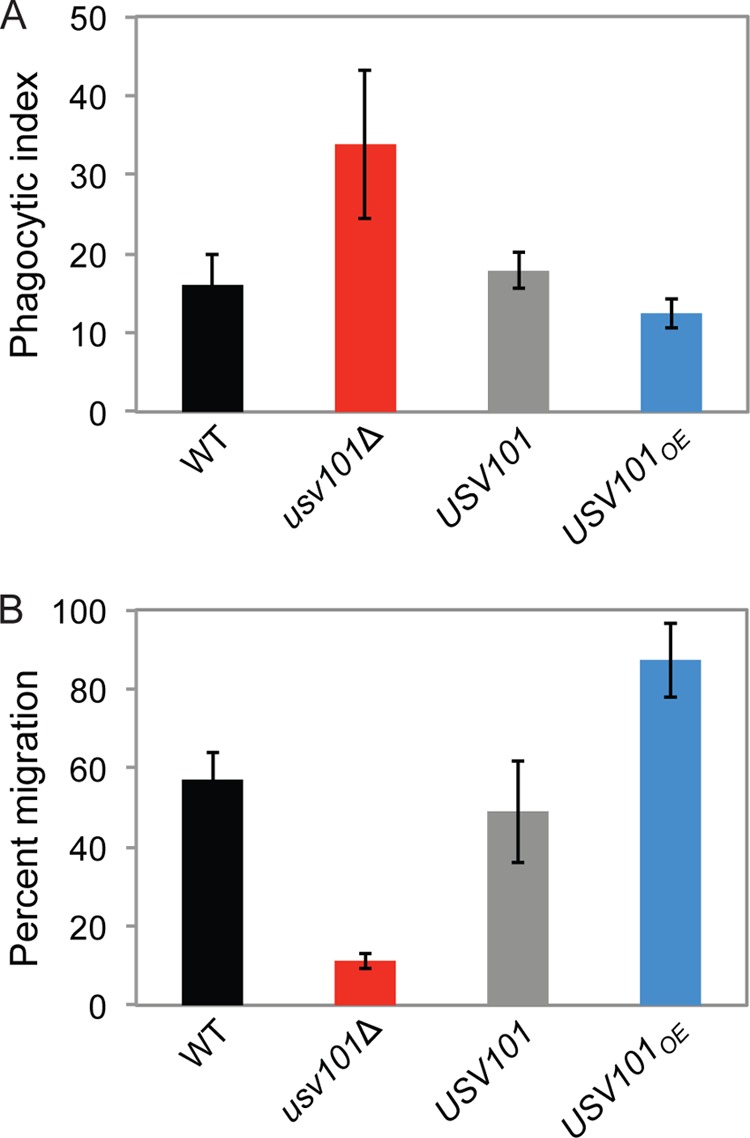
Host interactions are influenced by the levels of Usv101. (A) Phagocytic index (fungi per 100 THP-1 cells) for the indicated strains. (B) Percentage of serum-opsonized fungi of the indicated strains that migrate across model blood-brain barriers in 18 h. Means ± standard errors of the means (SEM) are shown.

The phenotypic changes in virulence traits exhibited by *usv101*Δ suggested that this strain would be altered in its interactions with host cells during infection. Critical in these interactions are phagocytes, which have a complex relationship with this facultative intracellular pathogen: while they oppose infection by destroying engulfed cryptococci and helping orchestrate antifungal host responses, they may also promote infection by providing a haven for the fungi in a hostile environment. Either of these outcomes, however, begins with engulfment of *C. neoformans*. To assess this interaction, we used an automated image-based assay to measure fungal uptake by human THP-1 cells (24). Surprisingly, the engulfment of *usv101*Δ cells was significantly higher (and that of *USV101_OE_* cells was lower) than those of wild-type and complemented controls (Fig. 3A). This was the opposite of what we expected, since larger capsules generally inhibit phagocytosis (24). Once internalized, however, viability of the internalized fungi was similar for all strains (not shown).

A second key host interaction occurs between *C. neoformans* and brain microvascular endothelial cells (BMECs) of the blood-brain barrier (BBB), which the pathogen must cross to cause meningoencephalitis. We assayed this transit *in vitro*, using model BBBs grown in Transwell plates. Notably, the *usv101*Δ strain was significantly impaired in BBB crossing compared to the wild type and the complemented strain (Fig. 3B); as with capsule size and uptake, the phenotype of the *USV101_OE_* strain in this assay differed from that of the wild type in the opposite direction, crossing into the brain more efficiently than wild type and the complemented mutant.

### Infection with the *usv101*Δ strain leads to delayed host response and death by pneumonia.

**FIG 4:**
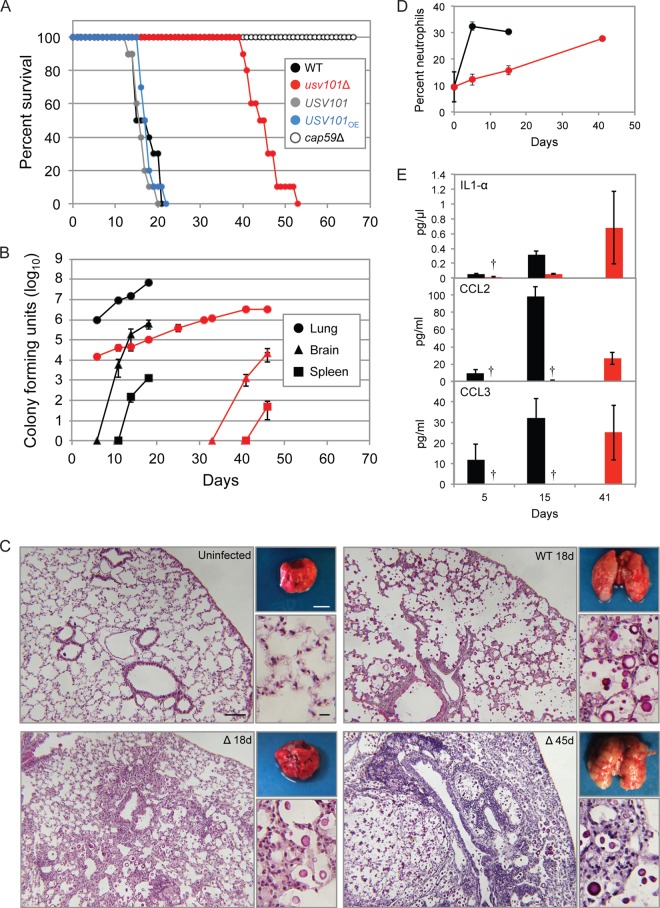
The *usv101*Δ mutant exhibits altered pathogenesis in a mouse model of cryptococcosis. A/JCr mice were intranasally inoculated with 5 × 10^4^ cells of the indicated strains. (A) Survival curve in which mice were monitored for weight loss with sacrifice triggered by weight below 80% of peak. (B) Mean ± SEM total CFU from the lungs, brains, and spleens of mice infected with the wild-type (black) or *usv101*Δ (red) *C. neoformans* strain. (C) Lungs of mice infected with *usv101*Δ and wild-type *C. neoformans* strains analyzed for gross pathology (top right; scale bar = 5 mm) or histology. Histological sections were stained with mucicarmine and imaged at 10× (larger image; scale bar = 100 µm) and 40× (bottom right; scale bar = 10 µm). WT, wild type; (Δ), *usv101*Δ. (D) Lung cells from mice infected with the wild-type (black) or mutant (red) *C. neoformans* strain were gated based on forward and side scatter, and CD11b^+^ Gr1^+^ cells were designated neutrophils. Shown are the means and standard deviations (SD) from three replicate analyses; differences between *usv101*Δ and wild-type strain-infected samples were significant (*P* < 0.0004 by Student’s *t* test) at 5 and 15 days postinfection. (E) Levels of the indicated immune mediators in lung homogenates (see Materials and Methods) at the times postinfection noted. The means ± SEM are plotted. Black, WT; red, *usv101*Δ mutant. Daggers indicate bars that are too small to be seen.

The reduced virulence of cryptococcal strains with decreased or absent capsule (25) has suggested that hypercapsular mutants would show increased virulence. Contrary to these expectations, however, we recently reported that multiple strains with enlarged capsule, including the *usv101*Δ mutant, are impaired in growth in short-term animal models of infection (8). These studies were performed with mice that were infected intranasally and then sacrificed 1 week later to assess lung burden: at this time of infection, *C. neoformans* has not yet disseminated to the brain at detectable levels. In these experiments, the lung burden of *usv101*Δ cells was almost 2 orders of magnitude lower than that of wild type at the 1-week time point (8), suggesting that the mutant strain would likely be cleared and consequently avirulent. To test this we performed a long-term virulence study, comparing the *usv101*Δ strain to its wild-type parent, KN99α, the *USV101* and *USV101_OE_* strains, and the acapsular (and avirulent) *cap59*Δ strain. As expected, all mice infected with the wild type, complemented mutant, and overexpression strains succumbed to the infection by 3 weeks postinoculation, while animals infected with the *cap59*Δ strain survived for the duration of the experiment (Fig. 4A). Surprisingly, although mice infected with the *usv101*Δ strain remained apparently healthy until week 6, they began to lose weight thereafter. Along with weight loss, these mice developed respiratory symptoms (rapid and labored breathing) but were otherwise alert and mobile; this behavior was in sharp contrast to that of wild-type-infected mice, which typically exhibit neurological symptoms (poor balance and lethargy) late in the course of infection. Together, these observations suggested a fundamental difference between the progression of disease caused by *C. neoformans* lacking Usv101 and the disease progression caused by the wild type.

To further probe the pattern of *usv101*Δ pathogenesis, we measured fungal burden over time in the lung, brain, and spleen of mice infected with the wild-type or *usv101*Δ mutant strain (Fig. 4B). At the earliest time point, 5 days after intranasal infection, the level of *usv101*Δ cells in the lung was already close to 100-fold lower than that of wild-type cells, consistent with our earlier study; this gap continued to increase until the wild-type-infected animals died. The mutant population in the lung continued to slowly grow, however, until those mice became ill, although it never reached the levels seen in wild-type infections despite the overt pulmonary symptoms. Also, although *usv101*Δ organisms eventually appeared in the brain, this was significantly delayed, as was dissemination to the spleen. For all three organs, the *usv101*Δ cell burden at the time of death was substantially lower than seen with wild-type fungi.

When harvesting organs for CFU analysis, we noticed that at the gross level, lungs collected from mice infected with wild-type fungi appeared more inflamed than those of mice infected with the *usv101*Δ mutant at 18 days postinfection, although by the time of death of the latter group (45 days), their lungs were dramatically enlarged and inflamed (Fig. 4C, insets). To pursue these observations, we examined pulmonary histology. Wild-type-infected animals at day 11 showed a uniform distribution of *C. neoformans* throughout the lung, most with large capsules, and mixed inflammation surrounding the airways, with predominantly polymorphonuclear leukocytes and some monocytic infiltrates (not shown). By day 18 (when WT mice succumbed to infection), increased inflammation and large numbers of fungi were evident throughout the lung (Fig. 4C, WT 18 days). In contrast to this disease progression in WT mice, *usv101*Δ strain-infected mouse lungs at day 11 showed overall fewer fungi (consistent with lower lung burden); these were centered on airways and surrounded by localized inflammation, with relatively few organisms observed peripherally in the lung (not shown). By day 18, increasing numbers of fungi could be seen, with a more diffuse distribution and inflammatory response but preservation of normal tissue appearance in some areas (Fig. 4C, Δ 18d). As infection progressed in *usv101*Δ strain-infected mice (day 32 [not shown]), the lesions became larger, extending to the periphery and occupying parenchymal spaces in the lung. Finally, by 6 to 7 weeks postinfection, when these animals succumbed to disease, most of the lung showed discrete lesions with concentrated organisms and exacerbated inflammation (Fig. 4C, Δ 45d). This is consistent with the respiratory distress of these animals.

When we probed the host response by profiling lung homogenates at various times after infection, we noted that neutrophil infiltration was significantly delayed in mice infected with the *usv101*Δ strain (Fig. 4D, red), compared to mice infected with the wild type (Fig. 4D, black). This observation was consistent with cytokine profiling of parallel lung samples (Fig. 4E), which showed similarly delayed induction of interleukin-1α (IL-1α), a proinflammatory cytokine that promotes neutrophil accumulation (26), as well as of neutrophil-attracting cytokines CCL2 (monocyte chemoattractant protein 1 [MCP-1]) and CCL3 (macrophage inflammatory protein 1α [MIP-1α]) (27).

### Usv101 repressor function.

To understand the striking phenotypes of cells lacking Usv101, we turned to its regulatory function. We have profiled virulence-associated traits (melanization, capsule size, capsule polysaccharide release, and pulmonary growth in mice) of 41 *C. neoformans* regulatory mutants in the same strain background (8). Of these strains, only the *usv101*Δ and *ada2*Δ strains showed defects in all of the features tested despite growing at rates similar to the wild type in multiple media (not shown). We previously combined network analysis and chromatin immunoprecipitation (ChIP) to define the regulatory network of Ada2 (7); here we determined how Usv101 regulates multiple virulence-associated traits.

**FIG 5:**
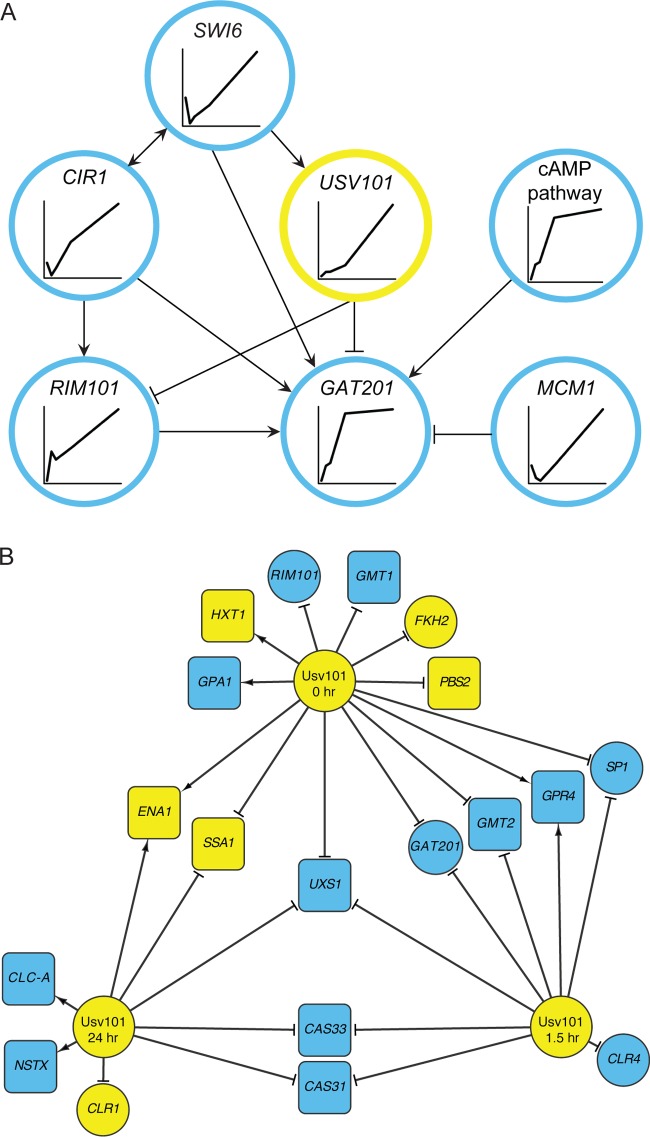
Regulatory interactions of Usv101. (A) Capsule-involved regulators of *USV101*, *GAT201*, or *RIM101* as determined by the NetProphet network (see Text S1 in the supplemental material) and their temporal expression patterns during 24 h of capsule induction. Targets of the node labeled “cAMP pathway” are regulated by both Pkr1 (the repressive subunit of the heterotetrameric complex) and Cac1 (the adenylate cyclase catalytic subunit) in opposite directions. Inside that node, the estimated activity of the cAMP pathway is plotted against time (see Text S1 for details). (B) Usv101 regulation of targets required for normal capsule growth. Shown are direct, functional targets of Usv101 under noninducing conditions (top center), 90 min after shifting to inducing conditions (bottom right), and 24 h after shifting to induction conditions (bottom left). All targets shown are required for normal capsule growth and either were bound by Usv101 in one of our ChiP-seq experiments or showed a strong potential for binding by Usv101 (based on their promoter sequences and the Usv101 binding motif we recovered from ChIP-seq). Targets that met those criteria are shown as being regulated at a given time if they were differentially expressed between wild-type and *usv101*Δ cells at that time point. Round nodes, target genes encoding TFs; square nodes, non-TF target genes; blue nodes, corresponding mutants are hypocapsular; yellow nodes, corresponding mutants are hypercapsular; arrowheaded lines, activation; T-headed lines, repression.

We first examined the regulatory context of Usv101 together with two other key capsule TFs: Gat201 (28), and Rim101 (29). We mapped regulators that both act on these 3 TFs and whose deletion influences capsule thickness (see Text S1 in the supplemental material for details). The resulting network (Fig. 5A) shows that *USV101* is positioned high in the capsule regulation cascade, regulated only by *SWI6*. This contrasts with *RIM101*, which has two regulatory inputs by capsule-implicated factors, and *GAT201*, which has six. Notably, *USV101* does not appear to be affected by the cyclic AMP pathway; instead its influence converges with that of the cyclic AMP (cAMP) pathway at *GAT201*. Finally, *USV101* is the only gene in this network whose deletion yields enlarged capsules. We also examined the kinetics of expression of the capsule-implicated TFs in this network (Fig. 5A, inset graphs). This showed that the variability in expression pattern occurred primarily at the early time points, with all mRNAs having their greatest expression at 24 h (see Text S1 for calculation of cAMP pathway activity).

To probe the regulatory function of Usv101 in detail, we next analyzed which capsule-implicated genes it regulates directly and how that target set changes during capsule induction. To do this we carried out RNA-seq experiments on the *usv101*Δ mutant and WT strains at three time points: before shifting to capsule-inducing conditions (0 min), 90 min after shifting (8), and 24 h after shifting. We identified the functional targets of Usv101 at each time point as those genes whose expression in the *usv101*Δ mutant was significantly different from their expression in WT (false discovery rate [FDR] of <0.02). We also carried out ChIP sequencing (ChIP-seq) experiments on cells expressing epitope-tagged Usv101 at both 0 and 90 min postinduction (8). We identified the direct functional targets of Usv101 at each time point as functional targets at that time point that were also ChIP positive at either 0 or 90 min postinduction. Notably, we were able to identify the same binding motif for Usv101 independently in both the ChIP-seq and the RNA-seq data. Using this motif, we scored all promoters according to their potential for binding Usv101 and considered those functional targets that scored in the top quintile as plausible direct functional targets. We then selected from the direct functional targets (identified by RNA-seq and either ChIP or binding potential) those whose corresponding deletion mutants had abnormal capsule. The result is shown in Fig. 5B.

Of the 19 direct, functional, capsule-involved targets shown in Fig. 5B, 13 are repressed by Usv101 (68%). When *all* direct, functional targets of Usv101 are considered, regardless of capsule involvement, there are fewer repressed targets under noninducing conditions (time zero of capsule induction), but the balance shifts sharply in favor of repression during the response to capsule-inducing conditions (see Fig. S3 in the supplemental material). These results support Usv101’s role as a transcriptional repressor that is central to capsule regulation.

### TF targets of Usv101.

**FIG 6:**
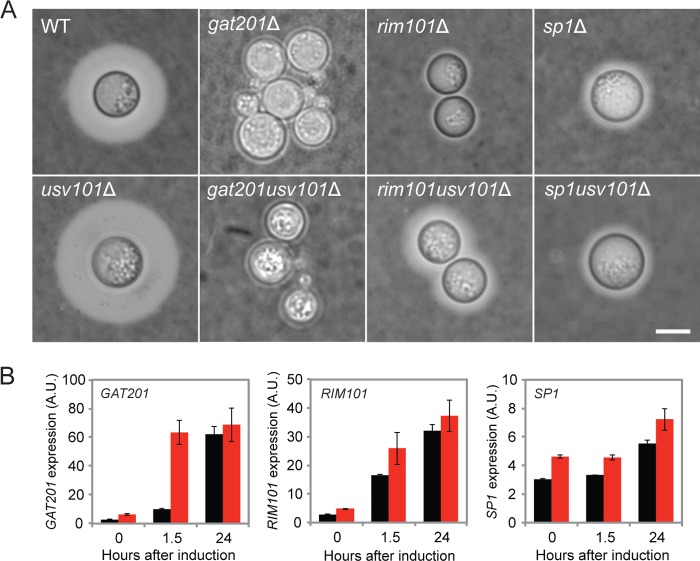
Major transcription factors that are regulated by Usv101. (A) The indicated strains were grown for 24 h under capsule-inducing conditions and stained with India ink for capsule visualization by light microscopy. All images are at the same scale (scale bar = 5 µm). Capsule thickness values for these strains are tabulated in Table S1B in the supplemental material. (B) Expression levels of *GAT201*, *RIM101*, and *SP1* in wild-type cells (black) and *usv101*Δ mutant cells (red) at 0, 1.5, and 24 h after shifting to capsule induction conditions, determined by RNA-seq (see Materials and Methods). A.U., arbitrary units. Means ± SEM are shown.

The targets of Usv101 include both transcriptional regulators (circles in Fig. 5B) and downstream proteins with other biological functions. The first group includes 3 TFs whose absence results in minor capsule thickness abnormalities (*FKH2*, *CLR4*, and *CLR1* [8]) and three others whose corresponding mutants have dramatically reduced capsule thickness (*GAT201*, *RIM101*, and *SP1* [28, 30–32]) (Fig. 6A). These genes show increased expression in response to capsule induction (Fig. 6B, black bars), consistent with a role in furthering capsule growth. Deletion of *USV101* increases the expression of all three (Fig. 6B, red bars), with the greatest effect on *GAT201* at 90 min after induction. Overexpression of *USV101* represses *GAT201* slightly at 90 min but has little effect on *RIM101* or *SP1* (see Table S2 in the supplemental material), suggesting that its repressive effect on the latter two genes is already saturated at WT levels.

To better understand the relationships between Usv101 and Gat201, we constructed and characterized a *gat201*Δ *usv101*Δ double mutant. This strain was severely hypocapsular, like the *gat201*Δ single mutant (Fig. 6A), showing that capsule formation requires factors whose expression depends on the presence of Gat201. However, we know that expression of many other Usv101 targets is also perturbed in this mutant (Fig. 5A). To uncouple changes in *GAT201* expression from the presence or absence of Usv101, we replaced the endogenous *GAT201* promoter with four other promoters, which we know from RNA-seq are independent of Usv101 (see Materials and Methods). We then analyzed the resulting strains for capsule thickness (see Table S1B in the supplemental material) and gene expression (by RNA-seq). We found that reducing *GAT201* expression, in the presence or absence of *USV101*, reduces capsule thickness (see Fig. S4 in the supplemental material), suggesting that increased *GAT201* expression contributes to the capsule expansion of *usv101*Δ mutants.

**FIG 7:**
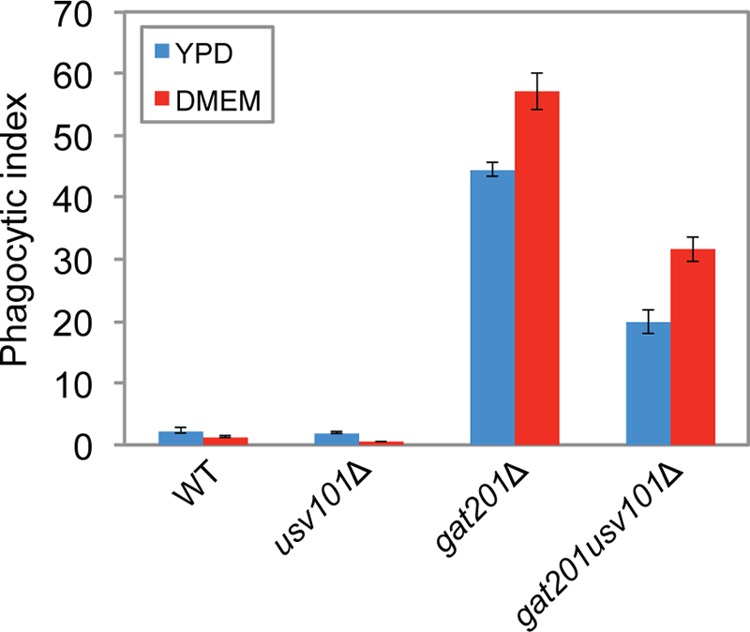
Macrophage uptake of unopsonized *gat201*Δ *usv101*Δ cells is between those observed with the *usv101*Δ and *gat201*Δ single mutants. The indicated strains (unopsonized) were incubated with macrophages for 4 h and processed to assess phagocytic index (fungi per 100 THP-1 cells). Cells were grown for 24 h in the presence (DMEM) or absence (YPD) of capsule-inducing conditions prior to uptake. Means ± SD are shown.

The capsule thickness phenotype of the *usv101*Δ strain depends on the presence of *GAT201*, such that the *gat201*Δ *usv101*Δ double mutant matches the *gat201*Δ single mutant, but this relationship does not hold for all phenotypes. For example, like Chun et al (32), we found that unopsonized wild-type cells are poorly engulfed by host phagocytes, while *gat201*Δ mutants are taken up avidly; we also noted that deletion of *USV101* has little effect on fungal engulfment in the wild-type background (Fig. 7). However, the *gat201*Δ *usv101*Δ double mutant, rather than resembling the *gat201*Δ single mutant, shows a phenotype midway between the low uptake of the wild-type and *usv101*Δ strains and the high uptake of the *gat201*Δ single mutant. (This may be because Usv101 and Gat201 regulate *BLP1* in opposite directions [8, 32].) A similar pattern is seen when the cells are induced to form capsules before exposure to phagocytes (Fig. 7, DMEM), even though the induced capsule sizes of the double mutant and *gat201*Δ single mutant are quite similar (and distinct from that of the wild type [Fig. 6B]). Finally, the *gat201*Δ *usv101*Δ double mutant is more impaired than either single mutant in capsule shedding (not shown). In summary, for some phenotypes, *USV101* deletion has an effect only in the presence of *GAT201*, for some it has an effect only in the absence of *GAT201*, and for some, the effects of the two deletions are relatively independent.

We next examined the relationship between Usv101 and its other 2 TF targets, Rim101 and Sp1, by deleting *USV101* in *rim101*Δ or *sp1*Δ mutants, both of which are hypocapsular. In combination with *rim101*Δ or *sp1*Δ, unlike with *gat201*Δ, *USV101* deletion increased capsule size (Fig. 6B; see Table S1B in the supplemental material). Consistent with the somewhat less severe capsule phenotype of the *rim101*Δ and *sp1*Δ mutants, this suggests that neither Rim101 nor Sp1 is strictly required for capsule formation or the capsule-expanding effects of *USV101* deletion.

### Usv101 target analysis elucidates the phenotypic changes in *usv101*Δ cells.

**FIG 8:**
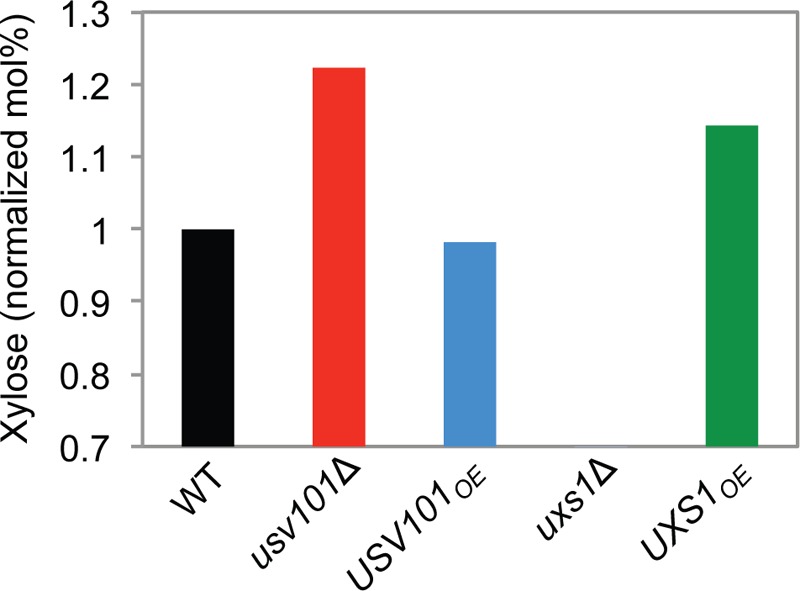
The xylose content of GXM is increased when Usv101 is absent or Uxs1 is overexpressed. GXM polysaccharide was isolated from the indicated strains, and monosaccharide composition was determined as detailed in Materials and Methods. Plotted is the xylose mole percent, normalized to wild-type values measured in parallel. Full compositional data are provided in Table S3 in the supplemental material. No xylose was detected in *uxs1*Δ cells.

We wished to extend our analysis of Usv101 to understand the mutant phenotypes mechanistically. To do this, we returned to our analysis of its target genes and assessed those direct targets that are not themselves DNA-binding proteins (squares in Fig. 5B). Only one capsule-involved target was significantly repressed by Usv101 at all time points we examined (Fig. 5B, center). This gene, *UXS1*, encodes a UDP-xylose synthase that decarboxylates UDP-glucuronic acid to form UDP-xylose (33). Both of these compounds are nucleotide sugars, activated molecules that act as donors of monosaccharides for glycan synthesis, and both provide major components of the dominant capsule polysaccharide, glucuronoxylomannan (GXM). In the absence of Usv101 *UXS1* would be derepressed, leading to an increase in UDP-xylose production. Consistent with this expectation, there is more xylose and less glucuronic acid in GXM of the *usv101*Δ strain (Fig. 8; see Table S3A in the supplemental material), compared to the wild type and the *USV101*-overexpressing strain. To directly test the effects of increased Uxs1 on capsule synthesis, we generated a strain in which *UXS1* is driven by the actin promoter. As predicted by our model, the *UXS1*-overexpressing strain was hypercapsular (see Table S1B in the supplemental material), and its GXM showed increased xylose content (Fig. 8; see Table S3B in the supplemental material), both phenotypes similar to those of *usv101*Δ cells. It was only slightly impaired in virulence, however, suggesting that factors beyond capsule composition and thickness impact the virulence of the *usv101*Δ strain (see Fig. S5 in the supplemental material).

Our computational analyses allowed us to link the absence of *USV101* to the observed changes in capsule size and composition via *UXS1.* We further probed our RNA-seq data for explanations of other characteristics we found to differ between wild-type and *usv101*Δ cells: melanin production, stress response, and cell wall thickness. Our expression analysis of genes implicated in melanin synthesis suggests that the defective melanization in *usv101*Δ cells (Fig. 2C) is likely due to significant downregulation of *CTR1* expression compared to wild type (see Table S4 in the supplemental material). This gene encodes the high-affinity copper transporter Ctr1 (34), formerly termed Ctr2 (35). Because copper is required for the catalytic function of laccase, which makes melanin (34), cells lacking *CTR1* do not produce this pigment. Furthermore, mice infected with *C. neoformans* lacking *CTR1* show reduced organ burden in both lung and brain, suggesting that reduced expression of this gene may also contribute to the altered pathogenesis of the *usv101*Δ strain*.*

Another phenotype we noted in *usv101*Δ cells was increased resistance to salt. Saline stress has been shown to increase transcription of a variety of genes in *S. cerevisiae*, including multiple solute transporters (36). Consistent with these observations, multiple cryptococcal genes annotated as monosaccharide transporters were significantly upregulated in the mutant compared to the wild type (see Table S4 in the supplemental material), potentially explaining its increased salt resistance.

Finally, *usv101*Δ cells showed significant changes in their expression of genes related to cell wall synthesis (see Table S4 in the supplemental material), which is critical for the display of virulence factors such as capsule and melanin and for host interactions. In particular, we observed reduced expression of three genes (*AGS1*, *CHS5*, and *SKN1*) whose products act in synthesis of major cell wall polysaccharides (alpha-glucan, chitin, and beta-glucan, respectively). The reduction in each case was greater than 2-fold and was highly statistically significant (see Table S4). Expression of a glucanase was also significantly increased, suggesting heightened cell wall remodeling, perhaps secondary to perturbations of cell wall synthesis. Finally, the gene encoding Gas1, which mediates beta-glucan rearrangement, was upregulated. This may be a compensatory change, by which the mutant attempts to strengthen its wall in the face of defective synthesis of individual components. Similarly, an increase in *CDA3* expression may reflect compensatory efforts to generate sufficient chitosan despite reduced synthesis of its precursor, chitin.

## DISCUSSION

Upon exposure to appropriate conditions, whether *in vitro* or in the context of mammalian infections, *C. neoformans* exhibits a dramatic expansion of its polysaccharide capsule. This process, which significantly impacts fungal virulence, requires both mRNA and protein synthesis (Fig. 1). To understand capsule synthesis and regulation in *C. neoformans*, we have applied a combination of computational and molecular techniques. The success of our strategy demonstrates the utility of computational modeling for defining mechanisms of microbial pathogenesis. Interweaving modeling with traditional methods of pathogenesis research has enabled us to discover numerous new transcriptional regulators and probe their activities and interactions (see references 7 and 8 and this work).

Our computational analyses led us to a C_2_H_2_ zinc finger transcription factor, Usv101, which participates in the control of multiple major virulence factors: capsule thickness, capsule shedding, and melanization. The Bahn group recently reported phenotypes of a large collection of TF mutants in *C. neoformans*, including *usv101*Δ (10). A number of their findings are consistent with our former (8) and current studies, including hypercapsularity and defects in melanin production and in virulence, which they measured in both an insect model and a short-term competitive growth assay in mice. A few traits of their mutant differed from ours, however, such as mating ability (which appeared normal in our mutant) and sensitivity to SDS and peroxide. It may be that some of these discrepancies are due to the different strain backgrounds used (H99 and KN99, which are known to differ in mating capacity [37]).

Expression of *USV101* increases in response to capsule-inducing conditions, under which Usv101 acts primarily as a repressor. Among its targets are three major regulators of capsule thickness: Gat201, Rim101, and Sp1. Lack of any of these regulators yields severely reduced capsules under conditions where wild-type capsules are large (Fig. 6B). (A previous study [31] showed that the *sp1* deletion mutant had enlarged capsule under certain conditions, but those conditions do not induce capsule in wild-type cells.) The fact that Usv101 represses these three positive regulators of capsule thickness under noninducing conditions and early in capsule induction suggests that much of the increase in capsule thickness in the *usv101*Δ mutant results from increased early expression of one or more of them along with increased expression of *UXS1* (discussed below). Furthermore, the increase in capsule thickness in the *usv101*Δ mutant depends on the presence of *GAT201* but not *RIM101* or *SP1*. However, the network linking these capsule regulators is not linear, as exemplified by the feed forward loop in which Usv101 represses *GAT201* both directly and via *RIM101* (Fig. 5A). This complexity is reflected in other virulence-related phenotypes, where the relationships between double and single mutants vary: for engulfment by macrophages, deletion of *USV101* moderates the effect of *GAT201* deletion, while in capsule shedding, the double mutant phenotype is more extreme than that of either single deletion.

Our results also suggest a complete causal model in which Usv101 directly represses *UXS1* (Fig. 5B), which encodes a UDP-xylose synthase that is required for capsule formation and virulence (38, 39). By increasing *UXS1* expression, deletion of *USV101* increases the xylose content of capsule (see Table S3A in the supplemental material), changing its morphology (Fig. 2) and the progression of disease (Fig. 4). Usv101’s direct repression of 3 TFs required for capsule growth (*GAT201*, *RIM101*, and *SP1*) early in the process of capsule induction is also likely to contribute to the increased capsule thickness of the *usv101*Δ mutant.

Mice infected with the *usv101*Δ mutant showed reduced fungal burdens in all tissues tested and a slowed increase in numbers of fungi over the course of infection. One factor in these observations could be altered growth rate of the mutant, although we observe only a subtle defect in *usv101*Δ mutant growth on rich medium and none in minimal yeast medium or RPMI. Another feature of the mutant that may affect the kinetics of infection is its increased engulfment by host phagocytes. This contrasts with the typical reduced phagocytosis of cells with large capsules and may be related to the altered composition of capsule polysaccharides that we have observed (see Table S3A in the supplemental material). Increased engulfment and altered cell wall of the mutant may also lead to its increased destruction, which would contribute to reduced burden. Finally, we found that *usv101*Δ cells are significantly impaired in their ability to cross the blood-brain barrier.

It is likely that decreased brain entry coupled with slowed growth and increased phagocytosis of the *usv101*Δ mutant delays disease progression and initially allows mutant-infected mice to survive. The reduced numbers and increased uptake of the fungi also result in a muted and delayed host response in comparison to wild-type-infected animals in the short term. This coincides with decreased early neutrophil accumulation and decreased induction of early cytokines in the lungs of *usv101*Δ strain-infected mice. However, the continuing infection eventually leads to an exacerbated pulmonary inflammatory response, likely due to altered early events in host immune response, which ultimately leads to the animals’ demise. The altered distribution and kinetics of *usv101*Δ accumulation in mice thus drastically change the progression of disease, both extending the time to death and changing the apparent cause of death from fungal meningitis to respiratory distress secondary to massive pulmonary inflammation. This paradoxical situation of the host response being more detrimental than the pathogen itself is reminiscent of immune reconstitution inflammatory syndrome (IRIS), which arises when HIV-positive patients with cryptococcal infection are treated with antiretroviral therapy and then respond energetically to the infection (40). This condition, also encountered in the context of infections with mycobacteria, *Pneumocystis*, and several viruses, presents significant therapeutic challenges (41). Notably, although the closely related pathogen *Cryptococcus gattii* also affects both the central nervous system (CNS) and lung, lung disease predominates significantly more often in both animal studies and in human populations.

Our computational analysis guided us to a major regulator of cryptococcal virulence, Usv101. Here, by combining additional computational analyses with *in vitro* and *in vivo* experimentation, we were able to predict and validate regulatory interactions. These interactions explain the increased capsule thickness of *usv101*Δ mutants and the lack of capsule on *usv101*Δ *gat201*Δ double mutant, as well as multiple virulence-related phenotypes that we observe in the mutant. Finally, we have observed that cells lacking Usv101 cause a novel pathogenic profile in mouse infection due to a delayed but exuberant host immune response, transforming the usual picture of lethal meningitis to one of fatal pneumonia. These approaches and observations are likely to apply to additional pathogenic microbes.

## MATERIALS AND METHODS

### Ethics statement.

All animal studies were reviewed and approved by the Animal Studies Committee of the Washington University School of Medicine and conducted according to the National Institutes of Health guidelines for housing and care of laboratory animals.

### Materials.

All chemicals and PCR primers were from Sigma-Aldrich, reagents and enzymes were from Life Technologies, and DNA cleanup kits were from Qiagen unless otherwise noted.

### Strains and growth conditions.

All strains used in this study were constructed in the serotype A strain KN99α (37), as detailed in Text S1 in the supplemental material. Unless noted otherwise, cells were grown with continuous shaking (230 rpm) at 30°C in YPD medium or at 30°C on agar plates. As appropriate, media were supplemented with either 100 μg/ml of nourseothricin (Werner Bioagents) or 100 μg/ml of Geneticin.

To induce capsule formation, cells cultured overnight in YPD were collected by centrifugation, washed in Dulbecco’s modified Eagle’s medium (DMEM), and adjusted to 10^6^ cells/ml in DMEM preconditioned to 37°C and 5% CO_2_ (capsule-inducing conditions) in 24-well plates. For inhibitor experiments, induction was carried out in the presence of either 20 μg/ml CHX or 200 μg/ml PHN as noted in Fig. 1. For CHX studies, viability was 63% after 8 h of drug treatment, decreasing to 38% at 24 h: if the drug was washed out at 8 h, viability recovered to 72% by 24 h of incubation.

### Strain phenotyping.

Details of the assays used to characterize the engineered strains are provided in Text S1 in the supplemental material. These include measurement of capsule thickness after India ink staining, quantitation of shed capsule by enzyme-linked immunosorbent assay (ELISA), determination of capsule polysaccharide composition, and assessment of melanin formation and release.

### Macrophage uptake and survival.

Engulfment of cryptococcal cells by human THP-1 macrophages was measured by high-content imaging as reported in reference 24 and detailed in Text S1 in the supplemental material. Fungal survival within THP-1 cells was assessed by counting colony-forming units (CFU) released from lysed cells at various times (see Text S1).

### BBB transmigration assays.

To measure fungal transversal of the blood-brain barrier (BBB), we added washed fungi to *in vitro* model BBBs that were generated and assessed for transendothelial electrical resistance (TEER) as described in reference 42 and then monitored fungal transmigration by CFU (for details, see Text S1 in the supplemental material).

### Infection studies.

Groups of 4- to 6-week-old female A/Jcr mice (National Cancer Institute) were intranasally inoculated with 5 × 10^4^ fungal cells and monitored for long-term survival and organ burden as detailed in Text S1 in the supplemental material. The methods used for histology, flow cytometry, and cytokine analysis of harvested lungs are also provided in Text S1.

### RNA isolation and RNA-seq.

Details of cell growth for RNA isolation, the isolation procedure, and preparation of RNA-seq libraries are provided in Text S1 in the supplemental material. Three biological replicates of each deletion mutant were profiled. To control for batch effects, a set of three wild-type replicates was profiled with every batch of deletion mutants. The wild-type replicate set was carried through the experimental stages, from induction to sequencing, at the same time as its matched mutant replicate sets. For all RNA-seq samples, the mean and median sequencing depth were 5.0 and 4.7 million reads, respectively, and the interquartile range of sequencing depth was 4.1 to 5.3 million reads. Details of data analysis and quality control are provided in Text S1 in the supplemental material.

### Computational methods.

The methods used to construct a network depicting the capsule-implicated putative direct functional targets of Usv101, to estimate the Usv101 binding potential on each gene’s promoter, to construct the network of regulators of *USV101*, *GAT204*, and *RIM101*, and to characterize TF mRNA level and activity are detailed in Text S1 in the supplemental material.

### Microarray data accession numbers.

All generated RNA-seq and ChIP-seq data have been submitted to the NCBI Gene Expression Omnibus (GEO; http://www.ncbi.nlm.nih.gov/geo/) under accession no. GSE69532 and GSE60398.

## SUPPLEMENTAL MATERIAL

Text S1  Supplemental methods. Download Text S1, PDF file, 0.2 MB

Figure S1  Relative expression of *USV101* plotted against capsule thickness for cells grown under a variety of different capsule-inducing conditions (blue symbols) (7) and the line that best fits the data (red). The fraction of variance in capsule size explained by the expression level of *USV101* (*R*^2^ = 0.52) was similar to that of *SSN801* (*R*^2^ = 0.55), a transcription factor gene whose deletion produces a hypercapsular phenotype. Download Figure S1, PDF file, 0.2 MB

Figure S2  Phenotypes of *usv101*Δ cells and controls. (A) Capsule polysaccharide shed from equal numbers of wild-type (WT), *usv101*Δ, complemented *usv101*Δ (*USV101*), and *USV101-*overexpressing strains (*USV101_OE_*) was quantitated by ELISA (see Materials and Methods). Means ± SEM are plotted for results from two independent experiments with duplicate samples. (B) The indicated strains were grown overnight in YPD and then diluted into either the same medium or l-DOPA medium (to induce melanin formation) and incubated for an additional 20 h. Serial dilutions were then plated on YNB or the same medium supplemented with NaNO_2_, and plates were incubated for 2 days at 37°C. Download Figure S2, PDF file, 1.7 MB

Figure S3  Percentage of Usv101’s direct targets that are repressed (blue, left-hand scale) and the concentration of *USV101* mRNA (red, right-hand scale) at 0, 1.5, and 24 h after shifting to capsule induction conditions. Download Figure S3, PDF file, 0.2 MB

Figure S4  Capsule thickness as a function of *GAT201* expression (determined by RNA-seq). Data are derived from strains in which the *GAT201* promoter was replaced by the promoters of four other genes (A through D, see Materials and Methods). Values for wild-type (WT), *usv101*Δ, and *gat201*Δ cells are also shown. Download Figure S4, PDF file, 0.4 MB

Figure S5  Fold-increase in colony-forming units (CFU) of the indicated strains in lung homogenates 1 week after intranasal inoculation of 6-week-old female C57/Bl6 mice, assessed as in reference 8. The means and standard errors of the means are plotted for wild-type mice (black bar [compiled from 8 independent experiments with 6 mice each and 1 experiment with 8 mice]) and for the indicated strains (groups of 8 mice). *, *P* ≤ 10^−2^, and **, *P* = 10^−7^, compared to the wild type (Student’s unpaired *t* test). Download Figure S5, PDF file, 0.2 MB

Table S1  Capsule thickness of cell populations. Table S1, PDF file, 0.1 MB

Table S2  Gene expression in cells overexpressing *USV101* Table S2, PDF file, 0.1 MB

Table S3  Monosaccharide composition of GXM from the indicated strains in mole percent. Components with values of ≤0.1 are not shown. Table S3, PDF file, 0.1 MB

Table S4  Fold change in gene expression of the *usv101*Δ mutant compared to the wild type. Table S4, PDF file, 0.1 MB
