# Supplementary material for: Computational Analysis Reveals a Key Regulator of Cryptococcal Virulence and Determinant of Host Response
Source: mBio. 2016 Apr 19;7(2):e00313-16. doi: 10.1128/mBio.00313-16 (PMC4850258; doi:10.1128/mBio.00313-16)
Supplement: Text S1 — Supplemental methods. Download [file mbo002162760s1.pdf]

SUPPLEMENTAL MATERIAL FOR:

Computational analysis reveals a key regulator of cryptococcal virulence and determinant of host response

Stacey R. Gish[1], Ezekiel J. Maier[2,3], Brian C. Haynes[2,3,6], Felipe H. Santiago-Tirado[1], Deepa L. Srikanta[1], Cynthia Z. Ma[3], Lucy X. Li[1], Matthew Williams[1], Erika C. Crouch[4], Shabaana A. Khader[1], Michael R. Brent[2,3,5,*], and Tamara L. Doering[1,*]

[1]Department of Molecular Microbiology, Washington University School of Medicine, St. Louis, MO 63110 USA

[2]Center for Genome Sciences & Systems Biology, Washington University School of Medicine, St. Louis, MO 63110 USA

[3]Department of Computer Science and Engineering, Washington University, St. Louis, MO, 63130

[4]Department of Pathology and Immunology, Washington University School of Medicine, St. Louis, MO 63110 USA

[5]Department of Genetics, Washington University School of Medicine, St. Louis, MO 63110 USA

[6]Current address for BCH is Asuragen, Inc., Austin, TX 78744

*Corresponding authors

Tamara L. Doering: doering@wustl.edu, 314-747-5597

Michael R. Brent: brent@wustl.edu, 314-286-0210

SUPPLEMENTAL METHODS

Strain construction

The *C. neoformans* H99 reference sequence was accessed through the Fungal Genome Initiative database at the MIT Broad Institute (http://www.broadinstitute.org/annotation/genome/cryptococcus_neoformans/MultiHome.html) and cryptococcal genomic DNA was isolated as described[1]. For gene deletion a split-marker approach[2] with biolistic transformation[3] was used to replace specific genomic targets with either a nourseothricin (NAT) or geneticin (G418) resistance marker as in reference[4]; a similar strategy was used for complementation at the original locus (as in reference[5]) and for promoter replacement. For over-expression, the *ACT* promoter (as in reference[6]) was inserted immediately upstream of the *USV101* or *UXS1* coding sequences, preceded by the NAT resistance cassette. To modulate *GAT201* expression, we similarly inserted promoter regions of CNAG_02044 (termed promoter A), CNAG_00456 (B), CNAG_03437 (C), or CNAG_07442 (D) upstream of that gene (cloning details available on request). The *gat201*Δ *usv101*Δ double mutant strain was made by crossing single mutants on V8 medium[7]. The *rim101*Δ *usv101*Δ and *sp1*Δ *usv101*Δ double mutants were generated by gene deletion (as above) into the *usv101*Δ mutant[4]. Plasmids for complementation and over-expression were checked by DNA sequencing and all transformants were confirmed by drug selection and PCR.

Phenotyping

To test mutant growth under various stress conditions, cells cultured overnight in YPD were collected by centrifugation, adjusted to 2×10^6 cells/ml in PBS, and three 10-fold serial dilutions were prepared. To test oxidative and nitrosative stress sensitivity, 5-µl aliquots of the original cell suspension and each dilution were spotted onto solid YNB medium (0.67% w/v yeast nitrogen base without amino acids, 2% w/v glucose, 2% w/v agar, 25 mM sodium succinate, pH 4.0) containing either 0.5 mM hydrogen peroxide (H_2O_2) or 0.5 mM sodium nitrite ($NaNO_2$). For other studies, 5-µl aliquots were spotted onto solid YPD medium containing 1.2 M NaCl, 1.5 M sorbitol, 6% (v/v) ethanol, 0.01%

(w/v) sodium dodecyl sulfate (SDS), 0.05% (w/v) Calcofluor white (Fluorescent Brightener 28), 0.05% (w/v) caffeine, or 50 mM Tris (pH 8.8).

To measure capsule thickness, cells were induced for capsule as described above. Capsules were then visualized by negative staining with India Ink and a minimum of 100 randomly chosen cells were imaged with identical acquisition settings on a Zeiss Axioskop 2 MOT Plus wide-field fluorescence microscope for measurement of capsule thickness.

To quantitate shed capsule, we induced capsule (as above) for 90 min, removed the cells by centrifugation, and measured the GXM content of the supernatant fraction by ELISA as in reference [8]. To assess capsule monosaccharide composition, GXM was isolated from culture supernatant fractions and analyzed at the Complex Carbohydrate Research Center as in reference [9].

To assess shed melanin, cells were grown overnight in YPD, washed in water, resuspended at 10^6/ml in L-DOPA medium (per liter, 1 g L-asparagine, 1 g glucose, 3 g KH_2PO_4, 250 mg $MgSO_4$-$7H_20$, and 100 mg L-DOPA), and grown for 20 h at 30 °C with shaking (230 rpm). Triplicate samples were taken at 20 h, the cells pelleted, and the supernatant measured for OD_{475}. To assess cell-associated melanin, cells were spotted on solid L-DOPA medium (above ingredients with 1 mg/ml thiamine and D-biotin).

Macrophage uptake and survival

Engulfment of cryptococcal cells by human THP-1 macrophages was measured as in reference[10]. Briefly, *C. neoformans* strains were either grown in YPD or induced for capsule for 24 h as described above, collected by centrifugation, washed in PBS, stained with Lucifer Yellow dye, and, in some experiments, opsonized with 40% human serum (from healthy volunteers, obtained following a protocol approved by the Washington University School of Medicine IRB). Fungi were adjusted to 10^6 cells/ml, added to adherent THP-1 cells in a 96-well plate, and incubated for 1 h at 37°C in a 5% CO_2 atmosphere. Cells were fixed with 4% paraformaldehyde, stained with DAPI and CellMask™ Deep Red plasma membrane stain, and the plate was imaged on a Cytation 3 Cell Imaging Multi Mode Reader (BioTek, Winooski, VT). Images were analyzed using

the INCell Developer Toolbox (GE Healthcare) and the phagocytic index was calculated as ingested fungi per 100 macrophages.

To assess fungal survival within THP-1 macrophages, *C. neoformans* strains were grown overnight in YPD, washed in PBS, opsonized with 40% human serum, and 3.5 x 10^4 cells added to 3.5 x 10^5 THP-1 macrophages per well in a 12-well plate. After a 1 h incubation the plate was washed thoroughly with PBS and incubated for various time periods before the addition of 1 ml of lysis buffer (0.05% w/v SDS, 1 mM EDTA in dH_2O) per well. The lysate was diluted and plated on YPD agar to obtain CFUs.

Blood-brain barrier transmigration assays

To measure fungal traversal of the blood-brain barrier (BBB) we used *in vitro* model BBB as described in reference[11]. Briefly, 5 x 10^4 cells of the human cerebral microvascular endothelial cell line hCMEC/D3 were seeded on 12-well cell culture inserts (pore size 8 µm; BD Falcon, Corning) and grown to confluence (5 – 6 days), with media replaced on day 3. The integrity of the monolayer was monitored daily by measuring the transendothelial electrical resistance (TEER) with a EVOM2 voltohmmeter (World Precision Instruments) and was typically around 80 ohms*cm^2 at the day of the experiment. To assay transmigration, log-phase *C. neoformans* were washed twice in PBS and 10^8 cells were resuspended and incubated in 40% fresh human serum in PBS for 30 min. The cells were collected by centrifugation, resuspended at 2 x 10^6 cells/ml in migration media (RPMI containing 1% FBS), and 500 µls were used to replace the medium above the model barrier; migration medium alone was used to replace the medium in the lower chamber. At various times TEER was measured to confirm barrier integrity and 1-ml aliquots were removed from the bottom chamber and replaced with prewarmed media; the aliquots were analyzed for CFU as above.

Infection studies

Strains to be tested were cultured overnight in YPD medium, collected by centrifugation, washed in PBS, and diluted to 10^6 cells/ml in PBS for intranasal inoculation (50 µl) into 4-6 week-old female A/Jcr mice (National Cancer Institute) that had been anesthetized

with a combination of ketaset-HCl and xylazine. PBS alone was instilled as a control for histology (uninfected mice). Initial inocula were plated to confirm CFUs. To assess long-term survival, ten infected animals were weighed 1 h post-infection and at least every other day afterwards. Mice were sacrificed if their weight fell below 80% of peak (an outcome which in this protocol precedes any signs of disease) or upon completion of the study.

To follow organ burden, 30 mice were inoculated with *usv101*Δ and 12 with wild-type cells. The animals were weighed as above and three mice from each cohort were sacrificed every five days until completion of the study. If any mice fell to below 80% of peak weight, those animals were sacrificed, along with additional mice from the same group chosen at random to maintain a triplicate set. Lungs, brain, and spleen were harvested from all mice, homogenized in 5, 1, and 1 ml of PBS respectively, and serial dilutions of the homogenate plated on YPD agar for enumeration of colony forming units (CFU).

Histology

For histology lungs were perfused with 10% formalin (Sigma HT501128) via the right ventricle, harvested and stored in the same solution for 24 hours, and then soaked sequentially in PBS, 30% ethanol, and 50% ethanol before transfer to 70% ethanol and submission to the Histology Core Facility of Washington University School of Medicine for processing, embedding, sectioning, and staining.

Flow cytometry and cytokine analysis

Mice were inoculated as above with the wild type strain, *usv101*Δ, or PBS as an uninfected control. At 5, 15, and 45 days post-infection mice were sacrificed humanely and the lungs harvested. Pulmonary leukocytes were isolated as described in reference[12], adjusted to 5 x 10^6 cells/ml in PBS, and incubated with 10 µg/ml CD16/CD32 (Fc block™) (BD Biosciences, San Diego, CA) for 20 min at 4°C. They were then incubated in the dark for 20 min at 4 °C with a mixture of antibodies specific for CD19, CD11b, CD11c, CD14 (from BD Biosciences), and CD3, CD4, CD8a, and Gr-1 (from BioLegend, San Diego, CA), conjugated with the following dyes, respectively:

phycoerythrin (PE), allophycocyanin (APC), peridinin-chlorophyll protein (PerCP Cy5.5), APC Cy7, fluorescein isothiocyanate (FITC), PE Cy7, PE Cy5, and Brilliant Violet 421. Samples were washed twice with FACS buffer (2% fetal bovine serum, 0.05% NaN_3 in PBS) fixed with 2% paraformaldehyde in PBS, and analyzed on an LSRII flow cytometer (Benton Dickinson, Franklin Lakes, NJ) with data interpreted using FlowJo software (Tree Star Inc., Ashland, OR). Lung neutrophils were designated as CD11b+ Gr1+ cells.

For pulmonary cytokine analysis, lungs were placed into 2 ml PBS supplemented with cOmplete protease inhibitor cocktail (Roche; 1 tablet/10 ml), homogenized, and spun at 10,000 x *g* for 15 min at 4°C. The supernatant fraction was removed and 50 µl of each sample was used for analyte capture with the Cytokine Mouse Magnetic 20-Plex Panel Kit (Life Technologies). Cytokine levels were measured on the Luminex 100 with xPONENT 3.1 software (EMD Millipore, Billerica, MA).

RNA Isolation

Prior to each experiment, cells were transferred from -80°C to YPD agar plates and grown for 3 days before streaking for isolation. Cells (three biological replicates per strain) were then cultured from single colonies overnight in YPD and shifted to capsule-inducing conditions (DMEM, 37°C, 5% CO_2) for the desired interval prior to isolation of total RNA. To prevent mRNA degradation, ice-cold stop solution (5% Tris-saturated phenol in ethanol) was added directly to the cultures (1% v/v) before the approximately 2×10^8 cells were collected by centrifugation. The cells were suspended in TRIzol reagent and lysed by mechanical bead-beating at 4°C with 0.5-mm silica-zirconia beads for 3 min, followed by a 2-min rest on ice, for a total of 4 cycles. Following lysis, total RNA was extracted according to the manufacturer's instructions. Residual DNA was removed from the RNA preparation with the TURBO DNA-*free* kit.

RNA-seq library preparation and sequencing

Samples were isolated for RNA-seq library preparation as in reference[5]. Briefly, poly (A) RNA was selected from total RNA with the mRNA Catcher Plus Kit using an epMotion 5075 liquid handling robot (Eppendorf) and sheared by incubating in TURBO DNA-*free* buffer at 75 °C for 10 min. The samples were purified with the QIAquick PCR

Purification Kit, and first strand cDNA synthesis was performed using random hexameric primers and SuperScript III Reverse Transcriptase, followed by treatment with *E. coli* DNA ligase, DNA polymerase I, and RNase H for second-strand synthesis using standard methods. The cDNA libraries were end-repaired with a Quick Blunting kit and A-tailed using Klenow exo- with dATP (New England Biolabs). Illumina adapters with four base barcodes were ligated to the cDNA and fragments ranging from 150-250 bp in size were selected using gel electrophoresis. The libraries were enriched in a 10-cycle PCR using Phusion Hot Start II High-Fidelity DNA (Fermentas), purified, and pooled in equimolar ratios for multiplex sequencing on an Illumina HiSeq 2500.

RNA-seq design and data analysis

Three biological replicates of each deletion mutant were profiled. To control for batch effects, a set of three wild type replicates was profiled with every batch of deletion mutants. The wild type replicate set was carried through the experimental stages, from induction to sequencing, at the same time as its matched mutant replicate sets. For all RNA-seq samples, the mean and median sequencing depth were 5.0 and 4.7 million reads respectively and the interquartile range of sequencing depth was 4.1 - 5.3 million reads. Sequenced reads were aligned to the *C. neoformans* H99 reference sequence v2 using TopHat version 2.0.4[13] and Bowtie version 0.12.8[14]. Reads that aligned uniquely to the reference sequence were considered for gene expression quantification with Cufflinks version 2.0.2[15] and gene boundaries were defined by using version 2 of the *C. neoformans* genome annotation provided from the Broad institute. Gene expression was normalized using the Cufflinks option for upper-quartile normalization. After gene expression quantification, samples not passing the rigorous quality control filters described below were removed from consideration. Counts of reads mapping to each gene were quantitated using the htseq-count tool in version 0.5.3 of the HTSeq Python package[16,17]. Differential expression analysis comparing mutant and wild type expression profiles from matching conditions was performed using LIMMA[17,18] with the voom transformation applied to read counts[19]. All mutant and wild type expression profile sets from the same condition that passed the quality control standards were used for differential expression analysis. To account for batch effects between replicate sets,

a design matrix was constructed which considered batch and treatment effects. Genes were considered to have responded to the treatment factor if their q-value (false-discovery rate) was ≤ 0.05.

RNA-seq quality control

The mean and median expression of *USV101* in *usv101*Δ mutant expression profiles was 0.3% and 0% of *USV101* wild type levels respectively. The low levels of expression that remained for some samples was likely the result of errors in the sequencing process itself, either due to contamination between samples or a low level of error in matching barcodes for multiplexed samples to reads. The mean and median expression of *USV101* in $USV101_{OE}$ expression profiles was 801% and 844% of *USV101* wild type levels respectively. In addition, within each replicate set the median of all genes' coefficients of variation (CoV, the standard deviation divided by the mean) was required to be ≤ 0.2. Any replicate set which did not pass this CoV filter was not used for differential expression analysis, and the mutant expression profiles were remade. However, replicate sets were rescued if the median CoV could be lowered below 0.2 by removing an outlier expression profile replicate.

Computational methods (detailed by Figure)

Temporal expression of regulators of *USV101*, *GAT201*, and *RIM101*.

To construct the network of regulators of *USV101*, *GAT201*, and *RIM101* (Fig. 5A), we examined the 10,000 most confident predictions from our recently published network of capsule size regulators[4]. From among these, we selected the regulators that are required for normal capsule growth and are predicted to regulate *USV101*, *GAT201*, or *RIM101*. These regulators and the interactions among them are shown. For the cyclic AMP (cAMP) pathway, we combined the interactions involving Pkr1 (the repressive subunit of the heterotetrameric complex) and Cac1 (the adenylate cyclase catalytic subunit), keeping only those that were regulated in opposite directions by these two proteins.

With each regulator node in the network diagram, we plotted the temporal expression pattern of the regulator in WT cells at 0, 1.5, 3, 8, and 24 h after transfer to capsule-inducing conditions (reference[4]; GEO: GSE60398). Each plotted point is the median expression of the regulator at the indicated time in three RNA-seq replicates. To allow for each temporal expression pattern to be displayed on the same scale, the temporal expression pattern of each regulator was normalized to the 0-1 range by subtracting its minimum expression value from expression value at each time point and then dividing by its maximum expression value. We computed the time-course for the cAMP node by identifying the targets of cAMP that are predicted to be both activated by Cac1 and repressed by Pkr1. We inferred a cAMP temporal expression pattern as the median temporal expression pattern of the cAMP targeted genes.

Construction of the regulatory network map (Fig. 5B).

To construct a network depicting the capsule-implicated putative direct functional targets of Usv101, we identified the capsule involved regulatory targets of Usv101 in capsule non-inducing conditions (a.k.a. 0 hours post-induction) and in capsule inducing conditions at 1.5 hours and 24 hours post-induction. First, to identify the functional targets of Usv101 in each condition, we compared wild type and *usv101* mutant expression profiles from the same time point. Genes identified as differentially expressed (q-value ≤ 0.02) were considered functional targets of Usv101 at that time point. Next, the subset of functional targets of Usv101 at a given time point that are regulated directly by Usv101 were identified by intersecting the functional targets from each time point with a set of Usv101 direct physical targets. The set of Usv101 direct physical targets was defined as the union of 436 Usv101 ChIP-positive targets (reference[4]; GEO: GSE60398) and the top 20% of genes most likely to be bound by Usv101 according to binding potential estimates generated using the Usv101 position weight matrix (PWM) we inferred from our ChIP data (reference[4], Supplemental File 5). To estimate the Usv101 binding potential on each gene's promoter, FIMO[20] was used to scan the Usv101 ChIP-inferred PWM over the promoter of each *C. neoformans* gene, with promoters defined as the 600 bases upstream of the start codon. Binding sites that were identified by FIMO at a *P*-value ≤ 0.005 were considered in subsequent analyses.

Two models of binding were considered. For each TF, the strong site model ranks promoters containing one or more significant binding sites according to the negative log *P*-value of the most significant site. The weak site model ranks promoters containing one or more significant binding sites by the sum of the negative log *P*-values for all significant sites in a promoter. The final Usv101 binding potential score for each gene was computed using the geometric mean of the gene's strong and weak site model Usv101 binding potential scores.

Finally, only the 100 known capsule-implicated genes were considered for inclusion[4]. Therefore, at each time point, the known capsule-involved genes that are functional targets of Usv101, defined by differential expression analysis, and physical targets, defined by Usv101 ChIP support or PWM-based binding strength, are displayed.

GAT201, RIM101, and *SP1* expression.

The expression levels of *GAT201*, *RIM101,* and *SP1* in wild-type cells (Fig. 6A) were quantified by taking the median of the 3 replicates for the time-point in the time-course expression dataset (reference[4]; GEO: GSE60398). The expression of *GAT201* and *RIM101* in *usv101* mutants was quantified using RNA-seq expression profiles generated in this paper. Arbitrary units of gene expression were computed by dividing each RPKM gene expression value by 100,000.

Characterization of Usv101 mRNA level and activity (Figure S2).

The expression level of *USV101* was quantified by using an RNA-seq time course of WT cells immediately before transfer from rich media into capsule-inducing conditions and at 1.5, 3, 8, and 24 h after transfer (reference[4]; GEO: GSE60398). At each time, the expression level of *USV101* was computed as the median value of the three replicates. The expression profile of *USV101* across the time course was normalized by dividing the expression of *USV101* at each time point by the expression of *USV101* at the 1.5 hour time point.

The activity of Usv101 was also investigated by quantifying the number of putative direct functional targets for Usv101 in the non-inducing condition (time 0) and at 1.5 and 24 hours after transfer into capsule inducing conditions. At each time, the number of activated and repressed putative direct functional targets of Usv101 was determined by intersecting the ChIP-positive targets of Usv101[4] with the genes whose expression was at least 2-fold down-regulated or up-regulated, respectively, in the *usv101* mutant compared to wild type expression at the same time point.

References for Supplementary Methods

1 Nelson, R. T., Hua, J., Pryor, B. & Lodge, J. K. Identification of virulence mutants of the fungal pathogen *Cryptococcus neoformans* using signature-tagged mutagenesis. *Genetics* **157**, 935-947 (2001).

2 Fu, J., Hettler, E. & Wickes, B. L. Split marker transformation increases homologous integration frequency in *Cryptococcus neoformans*. *Fungal Genet Biol* **43**, 200-212 (2006).

3 Toffaletti, D. L., Rude, T. H., Johnston, S. A., Durack, D. T. & Perfect, J. R. Gene transfer in *Cryptococcus neoformans* by use of biolistic delivery of DNA. *Journal of Bacteriology* **175**, 1405-1411 (1993).

4 Maier, E. J. *et al.* Model-driven mapping of transcriptional networks reveals the circuitry and dynamics of virulence regulation. *Genome Res* **25**, 690-700 (2015).

5 Haynes, B. C. *et al.* Toward an integrated model of capsule regulation in *Cryptococcus neoformans*. *PLoS Pathog* **7**, e1002411 (2011).

6 Ory, J. J., Griffith, C. L. & Doering, T. L. An efficiently regulated promoter system for *Cryptococcus neoformans* utilizing the *CTR4* promoter. *Yeast* **21**, 919-926 (2004).

7 Kwon-Chung, K. J., Edman, C. & Wickes, B. L. Genetic association of mating types and virulence in *Cryptococcus neoformans*. *Infect. Immun.* **60**, 602-605 (1992).

8 Percival, A., Thorkildson, P. & Kozel, T. R. Monoclonal antibodies specific for immunorecessive epitopes of glucuronoxylomannan, the major capsular polysaccharide of *Cryptococcus neoformans*, reduce serotype bias in an immunoassay for cryptococcal antigen. *Clin Vaccine Immunol* **18**, 1292-1296 (2011).

9 Kumar, P. *et al.* Pbx proteins in *Cryptococcus neoformans* cell wall remodeling and capsule assembly. *Eukaryot Cell* **13**, 560-571 (2014).

10 Srikanta, D., Yang, M., Williams, M. & Doering, T. L. A sensitive high-throughput assay for evaluating host-pathogen interactions in *Cryptococcus neoformans* infection. *PLoS One* **6**, e22773 (2011).

11 Daniels, B. P. *et al.* Immortalized human cerebral microvascular endothelial cells maintain the properties of primary cells in an *in vitro* model of immune migration across the blood brain barrier. *J Neurosci Methods* **212**, 173-179 (2013).
12 Wozniak, K. L. & Levitz, S. M. Isolation and purification of antigenic components of Cryptococcus. *Methods Mol Biol* **470**, 71-83 (2009).
13 Trapnell, C., Pachter, L. & Salzberg, S. L. TopHat: discovering splice junctions with RNA-Seq. *Bioinformatics* **25**, 1105-1111 (2009).
14 Langmead, B., Trapnell, C., Pop, M. & Salzberg, S. L. Ultrafast and memory-efficient alignment of short DNA sequences to the human genome. *Genome Biol* **10**, R25 (2009).
15 Trapnell, C. *et al.* Transcript assembly and quantification by RNA-Seq reveals unannotated transcripts and isoform switching during cell differentiation. *Nat Biotechnol* **28**, 511-515 (2010).
16 Anders, S., Pyl, P. T. & Huber, W. HTSeq--a Python framework to work with high-throughput sequencing data. *Bioinformatics* **31**, 166-169 (2015).
17 Wettenhall, J. M. & Smyth, G. K. limmaGUI: a graphical user interface for linear modeling of microarray data. *Bioinformatics* **20**, 3705-3706 (2004).
18 Smyth, G. K. Linear models and empirical bayes methods for assessing differential expression in microarray experiments. *Statistical applications in genetics and molecular biology* **3**, Article3 (2004).
19 Law, C. W., Chen, Y., Shi, W. & Smyth, G. K. voom: Precision weights unlock linear model analysis tools for RNA-seq read counts. *Genome Biol* **15**, R29 (2014).
20 Grant, C. E., Bailey, T. L. & Noble, W. S. FIMO: scanning for occurrences of a given motif. *Bioinformatics* **27**, 1017-1018 (2011).
