# Supplementary figures and images for: Computational Analysis Reveals a Key Regulator of Cryptococcal Virulence and Determinant of Host Response
Source: mBio. 2016 Apr 19;7(2):e00313-16. doi: 10.1128/mBio.00313-16 (PMC4850258; doi:10.1128/mBio.00313-16)

Figure S1

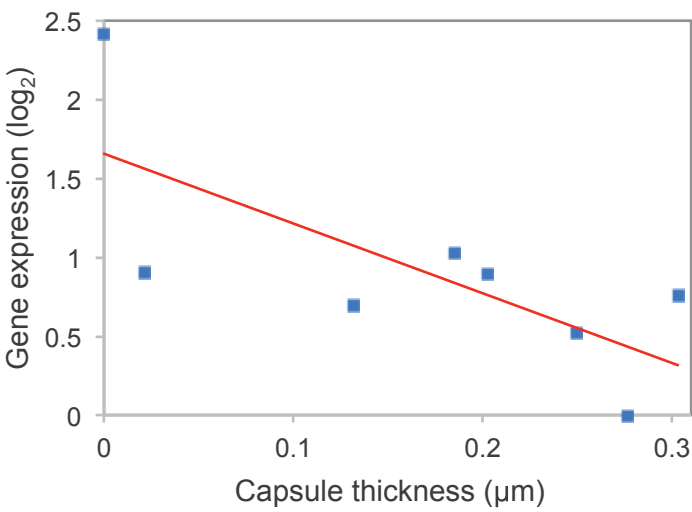

Supplement: Figure S1 — Relative expression of USV101 plotted against capsule thickness for cells grown under a variety of different capsule-inducing conditions (blue symbols) (7) and the line that best fits the data (red). The fraction of variance in capsule size explained by the expression level of USV101 (R2 = 0.52) was similar to that of SSN801 (R2 = 0.55), a transcription factor gene whose deletion produces a hypercapsular phenotype. Download [file mbo002162760sf1.pdf]

Figure S2

A

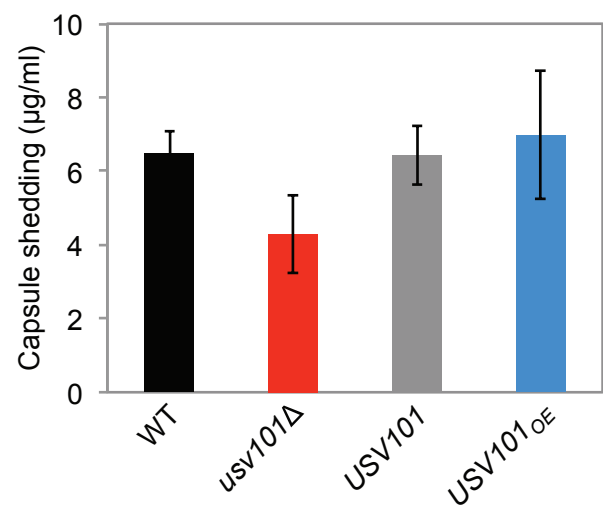

B

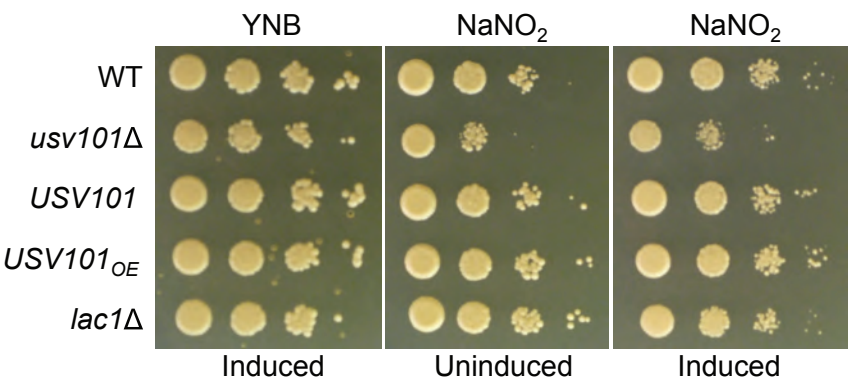

Supplement: Figure S2 — Phenotypes of usv101Δ cells and controls. (A) Capsule polysaccharide shed from equal numbers of wild-type (WT), usv101Δ, complemented usv101Δ (USV101), and USV101-overexpressing strains (USV101OE) was quantitated by ELISA (see Materials and Methods). Means ± SEM are plotted for results from two independent experiments with duplicate samples. (B) The indicated strains were grown overnight in YPD and then diluted into either the same medium or l-DOPA medium (to induce melanin formation) and incubated for an additional 20 h. Serial dilutions were then plated on YNB or the same medium supplemented with NaNO2, and plates were incubated for 2 days at 37°C. Download [file mbo002162760sf2.pdf]

Figure S3

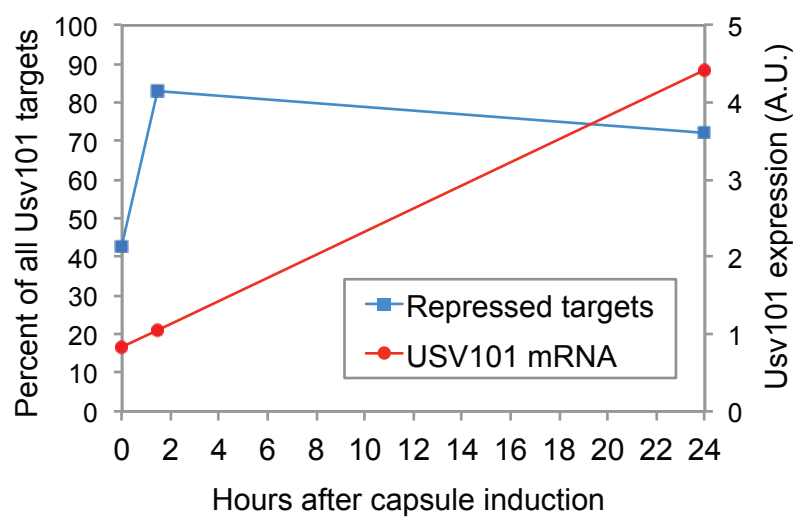

Supplement: Figure S3 — Percentage of Usv101’s direct targets that are repressed (blue, left-hand scale) and the concentration of USV101 mRNA (red, right-hand scale) at 0, 1.5, and 24 h after shifting to capsule induction conditions. Download [file mbo002162760sf3.pdf]

Figure S4

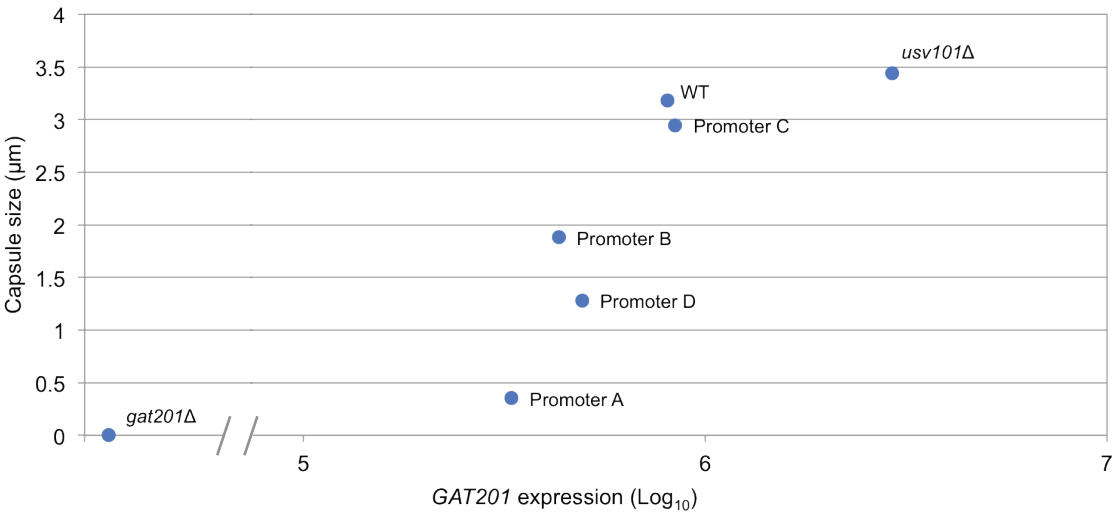

Supplement: Figure S4 — Capsule thickness as a function of GAT201 expression (determined by RNA-seq). Data are derived from strains in which the GAT201 promoter was replaced by the promoters of four other genes (A through D, see Materials and Methods). Values for wild-type (WT), usv101Δ, and gat201Δ cells are also shown. Download [file mbo002162760sf4.pdf]

Figure S5

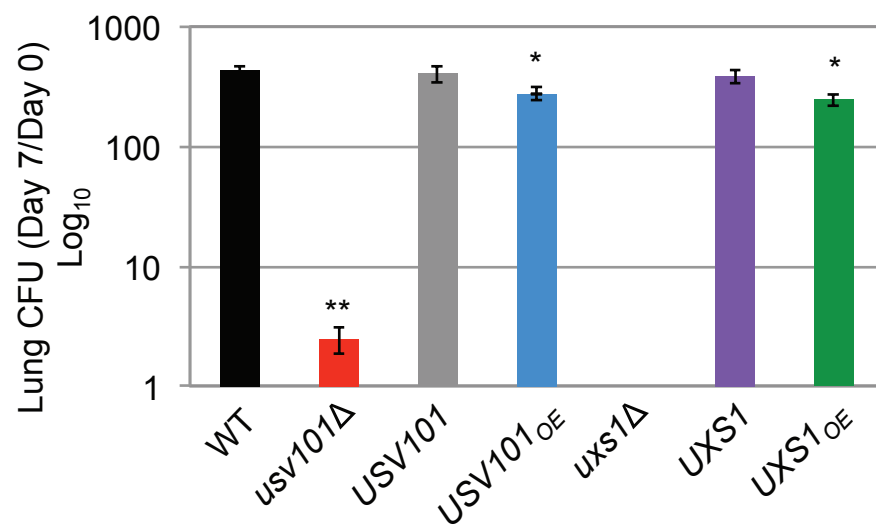

Supplement: Figure S5 — Fold-increase in colony-forming units (CFU) of the indicated strains in lung homogenates 1 week after intranasal inoculation of 6-week-old female C57/Bl6 mice, assessed as in reference 8. The means and standard errors of the means are plotted for wild-type mice (black bar [compiled from 8 independent experiments with 6 mice each and 1 experiment with 8 mice]) and for the indicated strains (groups of 8 mice). *, P ≤ 10−2, and **, P = 10−7, compared to the wild type (Student’s unpaired t test). Download [file mbo002162760sf5.pdf]
