# Supplementary material for: Computational Analysis Reveals a Key Regulator of Cryptococcal Virulence and Determinant of Host Response
Source: mBio. 2016 Apr 19;7(2):e00313-16. doi: 10.1128/mBio.00313-16 (PMC4850258; doi:10.1128/mBio.00313-16)
Supplement: Table S1 — Capsule thickness of cell populations. [file mbo002162760st1.pdf]

Table S1.

A. Capsule thickness after chemical treatment

Strain	Average size (μm)	±SEM
Control (−/−)	2.7	0.04
CHX (−/+)	1.9	0.05
CHX (+/+)	0.3	0.03
CHX (+/−)	3.0	0.07
Control (−/−)	2.7	0.04
PHN (−/+)	2.0	0.08
PHN (+/+)	0.4	0.03
PHN (+/−)	1.6	0.17

B. Capsule thickness of mutant strains

Strain	Average size, difference from wild type* (μm)	±SEM
WT	0	0.01
usv101Δ	0.4	0.02
USV101	-0.3	0.03
$USV101_{OE}$	-0.6	0.03
WT	0	0.01
uxs1Δ	-1.6	0.05
UXS1	0	0.05
$UXS1_{OE}$	0.3	0.05
WT	0	0.01
usv101Δ	0.4	0.02
gat201Δ	-2.4	0.03
gat201usv101Δ	-2.3	0.01
rim101Δ	-2.0	0.04
rim101usv101Δ	-0.9	0.04
sp1Δ	-2.1	0.03
sp1usv101Δ	-1.6	0.02
WT	0	0.01
usv101Δ	0.4	0.02
Promoter A	-2.8	0.03
Promoter B	-1.3	0.09
Promoter C	-0.2	0.05
Promoter D	-1.9	0.03

*Average for WT over all experiments was 3.1 microns
