# Supplementary material for: Computational Analysis Reveals a Key Regulator of Cryptococcal Virulence and Determinant of Host Response
Source: mBio. 2016 Apr 19;7(2):e00313-16. doi: 10.1128/mBio.00313-16 (PMC4850258; doi:10.1128/mBio.00313-16)
Supplement: Table S2 — Gene expression in cells overexpressing USV101 [file mbo002162760st2.pdf]

Table S2. Expression of selected TF genes upon induction

Strain	*GAT201* Expression (No induction)	±SEM	*GAT201* Expression (90 min post induction)	±SEM
WT	242,900	9,234	981,090	98,492
usv101Δ	640,143	63,437	6,353,790	845,063
$USV101_{OE}$	254,043	21,032	747,522	81,831
Strain	*RIM101* Expression (No induction)	±SEM	*RIM101* Expression (90 min post induction)	±SEM
WT	275,275	11,727	1,654,390	36,835
usv101Δ	481,585	22,259	2,585,010	551,578
$USV101_{OE}$	320,374	6,981	1,677,620	59,695
Strain	*SP1* Expression (No induction)	±SEM	*SP1* Expression (90 min post induction)	±SEM
WT	302,410	4,159	330,922	3,748
usv101Δ	464,113	11,359	455,577	16,359
$USV101_{OE}$	335,271	9,549	277,580	29,822
