# Supplementary material for: Computational Analysis Reveals a Key Regulator of Cryptococcal Virulence and Determinant of Host Response
Source: mBio. 2016 Apr 19;7(2):e00313-16. doi: 10.1128/mBio.00313-16 (PMC4850258; doi:10.1128/mBio.00313-16)
Supplement: Table S3 — Monosaccharide composition of GXM from the indicated strains in mole percent. Components with values of ≤0.1 are not shown. [file mbo002162760st3.pdf]

Table S3. GXM composition (mol %).

A. *USV101* strains

Strain	Xylose	Glucuronic Acid	Mannose
KN99α	33.6	11.9	54.6
usv101Δ	41.1	8.1	50.8
$USV101_{OE}$	33.0	10.1	56.8

B. *UXS1* strains

Strain	Xylose	Glucuronic Acid	Mannose
KN99α	25.8	7.0	67.2
uxs1Δ	-	10.0	90.0
UXS1	25.5	8.0	66.5
$UXS1_{OE}$	29.5	6.7	63.8
