# Supplementary material for: Computational Analysis Reveals a Key Regulator of Cryptococcal Virulence and Determinant of Host Response
Source: mBio. 2016 Apr 19;7(2):e00313-16. doi: 10.1128/mBio.00313-16 (PMC4850258; doi:10.1128/mBio.00313-16)
Supplement: Table S4 — Fold change in gene expression of the usv101Δ mutant compared to the wild type. [file mbo002162760st4.pdf]

Table S4. Gene expression in *usv101*Δ relative to WT *C. neoformans*

Locus	Name	Description	% of WT	pval*	Sc Homolog
**Melanization**					
CNAG_07701	*CTR1*	high-affinity copper transporter	49	$<10^{-3}$	*CTR3*
**Salt resistance**					
CNAG_02773	-	monosaccharide transporter	385	$<10^{-3}$	*STL1*
CNAG_02479	-	monosaccharide transporter	274	$<10^{-5}$	*STL1*
CNAG_05387	-	galactose transporter	255	$<10^{-11}$	*HXT5*
**Cell wall synthesis**					
CNAG_03120	*AGS1*	alpha-glucan synthase	49	$<10^{-2}$	*MAL12*
CNAG_05818	*CHS5*	chitin synthase	48	$<10^{-3}$	*CHS3*
CNAG_00897	*SKN1*	acts in beta glucan synthesis	49	$<10^{-2}$	*KRE6*
CNAG_00373	-	glucanase	330	$<10^{-4}$	*SPR1*
CNAG_06501	*GAS1*	glucanosyltransferase	259	$<10^{-4}$	*GAS1*
CNAG_01239	*CDA3*	chitin deacetylase	394	$<10^{-5}$	*CDA2*

*All p-values are adjusted for multiple testing
